# NY-ESO-1-Specific Circulating CD4^+^ T Cells in Ovarian Cancer Patients Are Prevalently T_H_1 Type Cells Undetectable in the CD25^+^FOXP3^+^Treg Compartment

**DOI:** 10.1371/journal.pone.0022845

**Published:** 2011-07-29

**Authors:** Nassima Redjimi, Karine Duperrier-Amouriaux, Isabelle Raimbaud, Immanuel Luescher, Danijel Dojcinovic, Jean-Marc Classe, Dominique Berton-Rigaud, Jean-Sébastien Frenel, Emmanuelle Bourbouloux, Danila Valmori, Maha Ayyoub

**Affiliations:** 1 Institut National de la Santé et de la Recherche Médicale, Unité 892, CLCC René Gauducheau, Saint Herblain, France; 2 Ludwig Institute for Cancer Research, Lausanne Branch, University of Lausanne, Epalinges, Switzerland; 3 Department of Surgery, CLCC René Gauducheau, Saint Herblain, France; 4 Department of Medical Oncology, CLCC René Gauducheau, Saint Herblain, France; 5 Faculty of Medicine, University of Nantes, Nantes, France; MRC National Institute for Medical Research, United Kingdom

## Abstract

Spontaneous CD4^+^ T-cell responses to the tumor-specific antigen NY-ESO-1 (ESO) are frequently found in patients with epithelial ovarian cancer (EOC). If these responses are of effector or/and Treg type, however, has remained unclear. Here, we have used functional approaches together with recently developed MHC class II/ESO tetramers to assess the frequency, phenotype and function of ESO-specific cells in circulating lymphocytes from EOC patients. We found that circulating ESO-specific CD4^+^ T cells in EOC patients with spontaneous immune responses to the antigen are prevalently T_H_1 type cells secreting IFN-γ but no IL-17 or IL-10 and are not suppressive. We detected tetramer^+^ cells *ex vivo*, at an average frequency of 1∶25000 memory cells, that is, significantly lower than in patients immunized with an ESO vaccine. ESO tetramer^+^ cells were mostly effector memory cells at advanced stages of differentiation and were not detected in circulating CD25^+^FOXP3^+^Treg. Thus, spontaneous CD4^+^ T-cell responses to ESO in cancer patients are prevalently of T_H_1 type and not Treg. Their relatively low frequency and advanced differentiation stage, however, may limit their efficacy, that may be boosted by immunogenic ESO vaccines.

## Introduction

CD4^+^ T-cell subsets play important and potentially opposite roles in tumor immunosurveillance [1], [2]. Type I helper (T_H_1) T cells, secreting the signature cytokine IFN-γ, favor the development of CD8^+^ cytolytic effectors (CTL), and mediate efficient anti-tumor responses. In contrast, regulatory/suppressor T cells (Treg), characterized *ex vivo* by high expression of the IL-2R α-chain (CD25) and of the lineage-specific transcription factor FOXP3 and low expression of the IL-7R α-chain (CD127), are believed to inhibit anti-tumor responses [3], [4], [5], [6], [7]. Treg, that fail to secrete IFN-γ or IL-2, have been reported to be present in increased proportions in cancer patients as compared to healthy individuals [8], [9]. Because the antigen specificity of Treg is largely unknown, it is unclear if the ability of Treg to inhibit anti-tumor responses is related or not to the presence/prevalence among them of tumor-antigen specific CD4^+^ T cells.

NY-ESO-1 (ESO), a tumor-specific antigen of the cancer/testis group frequently expressed in human tumors of different histological types, including ovarian cancers, but not in normal somatic tissues [10], [11], is a candidate for the development of generic anticancer vaccines [12]. ESO is highly immunogenic and elicits spontaneous humoral, CD4^+^ and CD8^+^ T-cell responses in patients bearing antigen-expressing tumors [11], [13], [14], [15]. In addition, ESO-specific antibody, CD4^+^ and CD8^+^ T-cell responses can be induced through immunization with ESO-based vaccines [16]. We have previously identified immunodominant regions recognized by ESO-specific CD4^+^ and CD8^+^ T cells [16] and have generated soluble fluorescent MHC class I and, recently, MHC class II/ESO peptide tetramers allowing the direct detection, phenotyping and isolation of ESO-specific T cells [17], [18]. Using MHC class II/ESO peptide tetramers to assess specific CD4^+^ T cells in patients immunized with a recombinant ESO protein administered with Montanide™ ISA 51 and GpG 7909, we have shown that vaccine-induced ESO-specific CD4^+^ T cells are prevalently T_H_1 cells, are detected *ex vivo* among memory (CD45RA^−^) cells, include both central memory (CCR7^+^) and effector memory (CCR7^−^) populations and do not include significant proportions of Treg [16], [17], [18]. Recent studies, however, have suggested that, in contrast to ESO-specific CD4^+^ T cells primed through vaccination, ESO-specific CD4^+^ T cells in patients with spontaneous immune responses may contain significant proportions of Treg [19] and that elevated proportions of circulating Treg in cancer patients may impair their responsiveness to ESO vaccines [20]. To address these concerns, in this study, we have used functional approaches, together with MHC class II/ESO peptide tetramers to assess ESO-specific cells among conventional and Treg CD4^+^ T-cell subsets in circulating lymphocytes of epithelial ovarian cancer (EOC) patients with detectable spontaneous immune responses to ESO.

## Results

### Assessment of memory conventional CD25^−^ and regulatory CD25^+^FOXP3^+^ CD4^+^ T-cell subsets in circulating lymphocytes of healthy donors and EOC patients

**Figure 1:**
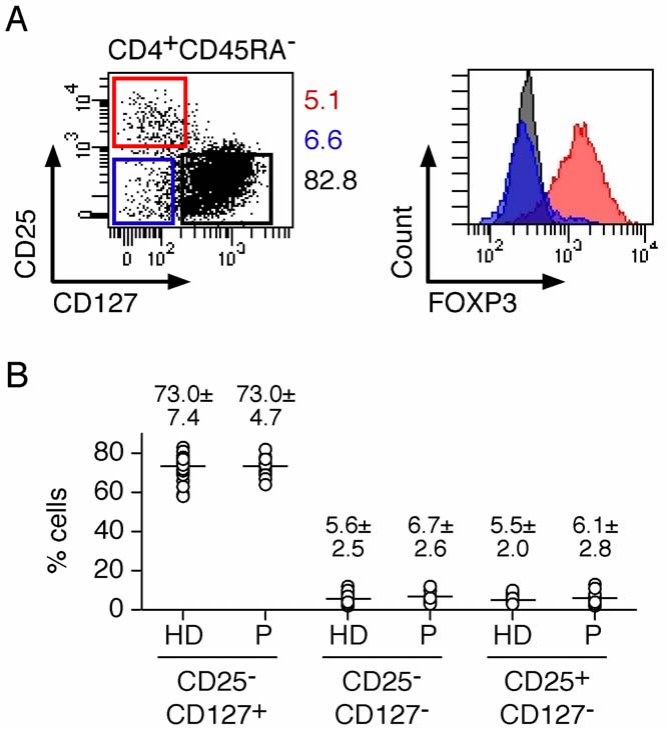
Phenotypic assessment of memory conventional and regulatory CD4^+^ T-cell subsets in circulating lymphocytes of healthy donors and EOC patients. **A**. CD4^+^ T cells were stained with anti-FOXP3, -CD25, -CD45RA and CD127 antibodies and analyzed by flow cytometry. Expression of CD25 and CD127 defines 3 populations of memory (CD45RA^−^) CD4^+^ T cells: conventional CD25^−^CD127^+^ and CD25^−^CD127^−^ and Treg CD25^+^CD127^−^ (left dot plot, numbers correspond to the proportion of each subset among memory CD4^+^ T cells). Histograms show the expression of FOXP3 in the defined memory CD4^+^ T-cell subsets. **B**. The proportion of conventional CD25^−^CD127^+^ and CD25^−^CD127^−^ and Treg CD25^+^CD127^−^ subsets, defined in **A**, among memory CD4^+^ T cells of healthy donors (HD, n = 27) and patients (P, n = 18).

**Figure 2:**
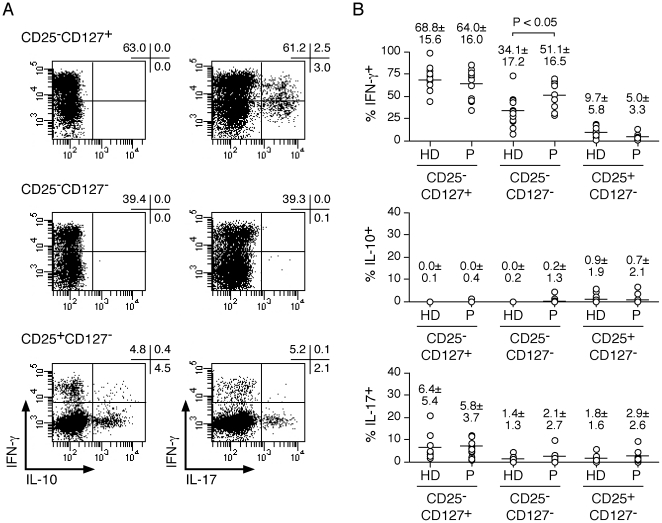
Functional assessment of memory conventional and regulatory CD4^+^ T-cell subsets in circulating lymphocytes of healthy donors and EOC patients. *Ex vivo*-sorted memory conventional, CD25^−^CD127^+^ and CD25^−^CD127^−^, and Treg, CD25^+^CD127^−^, populations from healthy donors (HD, n = 12) and patients (P, n = 12) were stimulated *in vitro* and day 12 cultures were assessed for IFN-γ, IL-10 and IL-17 production, following stimulation with PMA and ionomycin, in a 4-h intracellular cytokine secretion assay and analyzed by flow cytometry. Dot plots for one donor are shown in **A** and data for all healthy donors and patients are summarized in **B**. Statistical analyses were performed using a standard two-tailed *t*-test.

Among memory CD4^+^ T cells several subsets can be distinguished based on the expression of CD25 and CD127. Whereas conventional CD4^+^ T cells are CD25^−^CD127^+^, Treg are CD25^+^CD127^−^ and FOXP3^+^ (Figure 1A). A third population, CD25^−^CD127^−^, contains recently activated and IL-10-producing CD4^+^ T cells [21]. Whereas CD25^−^CD127^+^ cells are the majority of circulating memory CD4^+^ T cells, Treg and CD25^−^CD127^−^ populations are present in much lower and roughly equivalent proportions, representing each about 5%. Because previous reports have indicated that Treg populations can be increased in circulating lymphocytes from cancer patients as compared to healthy individuals, we compared the proportion of CD4^+^ T-cell subsets in circulating lymphocytes of EOC patients to healthy donors. We failed, however, to detect any significant differences in the proportion of circulating Treg in patients as compared to healthy donors (Figure 1B). Similarly, the proportion of CD25^−^CD127^−^ CD4^+^ T cells did not significantly differ between patients and healthy donors. To further characterize CD4^+^ T-cell subsets in circulating lymphocytes from EOC patients, we isolated them *ex vivo*, by flow cytometry cell sorting, stimulated them *in vitro* and assessed the cultures 12 days later for their capacity to secrete different cytokines. As expected, in both healthy donors and patients, CD25^−^ populations contained significantly higher proportions of cells secreting IFN-γ as compared to Treg (Figure 2). CD127^+^ populations contained higher proportions of IFN-γ-secreting cells than CD127^−^ populations. Interestingly, as compared to healthy donors, CD25^−^CD127^−^ populations from cancer patients contained higher proportions of IFN-γ-secreting cells. In contrast, the proportion of IL-17- or IL-10-secreting cells was not significantly different between healthy donors and patients for any of the populations.

### ESO-specific circulating CD4^+^ T cells from EOC patients with spontaneous immune responses are found in CD25^−^CD127^+^ and CD25^−^CD127^−^ populations but not in CD25^+^CD127^−^FOXP3^+^Treg and secrete IFN-γ but not IL-17 or IL-10

**Figure 3:**
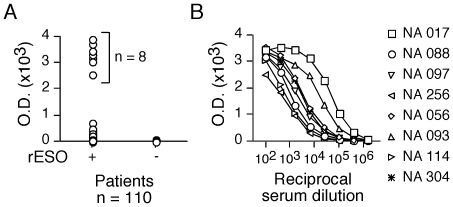
Assessment of ESO-specific antibody responses in sera of EOC patients. **A**. The presence of ESO-specific Ab in patients' sera was assessed by ELISA. Sera were initially assessed at a 1∶100 dilution on rESO-coated or on control uncoated plates. Sera were scored as ESO Ab^+^ if the optical density (OD) values obtained on rESO-coated plates were both at least 3 folds higher than those obtained for the same sample on uncoated plates and higher than the mean+6xSD of OD values obtained with sera from healthy individuals on rESO-coated plates (n = 53, mean+6xSD  = 750, data not shown). **B**. Serial dilutions of ESO Ab^+^ sera were tested on rESO-coated plates. Serum titer was calculated as the serum dilution yielding 50% of maximal OD.

**Figure 4:**
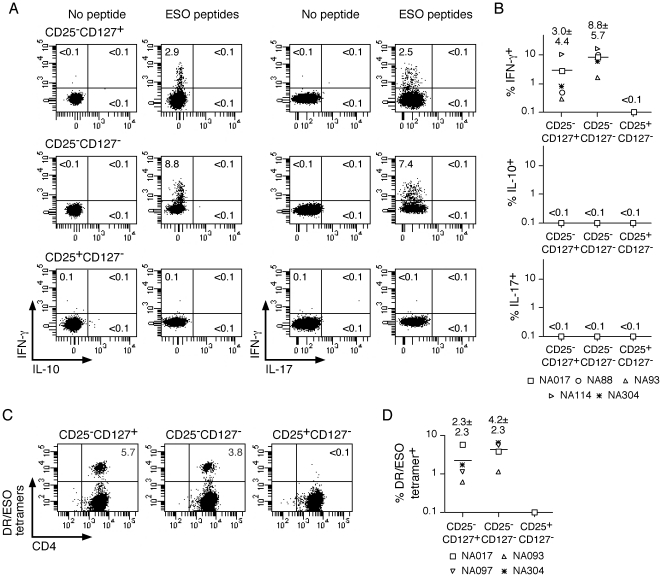
Assessment of ESO-specific cells in circulating memory CD4^+^ T-cell subsets of EOC patients. Memory conventional, CD25^−^CD127^+^ and CD25^−^CD127^−^, and Treg, CD25^+^CD127^−^, populations were sorted *ex vivo* from CD4^+^ T cells of ESO Ab^+^ patients and stimulated *in vitro* with a pool of long overlapping peptides spanning the full length ESO sequence. **A** and **B**. Day 12 cultures were assessed for IFN-γ, IL-10 and IL-17 production in a 4-h intracellular cytokine staining assay following stimulation in the absence or presence of the ESO peptide pool. Dot plots for one patient are shown in **A** and data obtained for all patients are summarized in **B**. **C** and **D**. Day 12 cultures were stained with DR52b/ESO_119–143_ (NA017, NA093 and NA097) or DR4/ESO_119–143_ (NA304) tetramers and anti-CD4 mAb and analyzed by flow cytometry. Dot plots for one patient are shown in **C** and data for all patients are summarized in **D**.

According to previous studies, about 40% of ovarian tumors express ESO and about 30% of EOC patients bearing ESO-expressing tumors develop specific spontaneous antibody (Ab) responses [22]. We assessed Ab responses to ESO in a cohort of 110 EOC patients (Table 1) in the patients' sera by ELISA as described [14]. We detected significant Ab responses to ESO in 8 (7%) of the patients (Figure 3A) at titers varying between 1∶500 and 1∶50000 (Figure 3B). Patients with detectable ESO Ab presented with high grade (II, III) tumors at stage III or IV and with serous histology (Table 1). For 6 patients, PBMC were available for analysis. To assess ESO-specific cells within defined circulating memory CD4^+^ T-cell subsets of these patients, we isolated them *ex vivo* by flow cytometry cell sorting, and stimulated them with a pool of long overlapping peptides spanning the ESO sequence, as described [16]. Twelve days later, we stimulated aliquots of the cultures with the ESO peptide pool and assessed specific cytokine production by intracellular staining. We detected significant proportions of cells producing IFN-γ in response to ESO in cultures from circulating CD25^−^CD127^+^ and CD25^−^CD127^−^ CD4^+^ T-cell populations, but not from Treg (Figure 4A and 4B). In contrast, we found only low proportions of cells secreting IL-10 or IL-17 in response to ESO in any of the populations. Together, these results indicated that the large majority of circulating ESO-specific CD4^+^ T cells in these patients were T_H_1 type IFN-γ-secreting cells, CD25^−^, both CD127^+^ and CD127^−^, and were not detectable in CD25^+^FOXP3^+^Treg.

**Table 1:** Patient characteristics.

	Total cohort	ESO Ab^+^
	110	8 (7%)
Age* [mean (range)], years	56 (31–76)	56 (46–67)
FIGO stage, n (%)		
I	29 (26)	
II	11 (10)	
III	59 (54)	6 (75)
IV	10 (9)	2 (25)
Undetermined	1 (1)	
Histology, n (%)		
Serous	63 (57)	7 (88)
Clear cell	11 (10)	
Endometriod	15 (14)	
Mucinous	4 (4)	
Undifferientiated	8 (7)	1 (12)
Others (mixed, carcinosarcoma)	7 (6)	
Undetermined	2 (2)	
Grade, n (%)		
1	15 (14)	
2	31 (28)	5 (63)
3	47 (43)	2 (25)
Undetermined	17 (15)	1 (12)

*Age at diagnosis.

### Assessment of ESO-specific T cells in CD4^+^ subsets using MHC class II/ESO peptide tetramers

**Figure 5:**
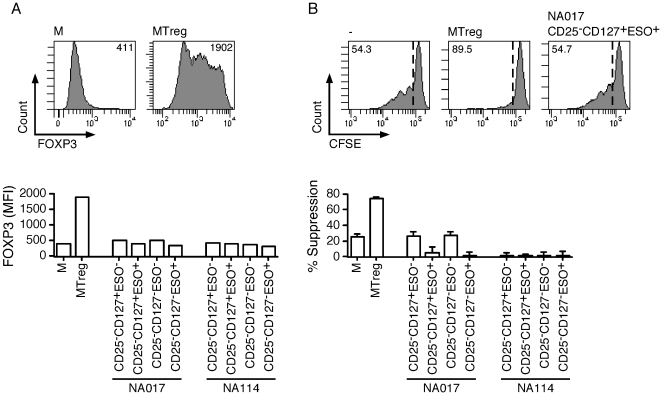
Assessment of the suppressive activity of ESO-specific CD4^+^ T-cell polyclonal populations. ESO-specific cells were isolated from peptide-stimulated CD25^−^CD127^+^ and CD25^−^CD127^−^ CD4^+^ T-cell cultures (Figure 4) by tetramer-guided (NA017) or IFN-γ-guided (NA114) flow cytometry cell sorting and expanded *in vitro*. The resulting polyclonal cultures contained >80% ESO-specific cells. **A**. ESO-specific polyclonal cultures as well as polyclonal cultures from the ESO^−^ fraction and control cultures of *in vitro*-expanded conventional memory CD4^+^ T cells (M) and memory Treg (MTreg), isolated *ex vivo* from healthy individuals, were stained with FOXP3-specific mAb and analyzed by flow cytometry. Numbers in dot plots correspond to the mean fluorescence intensity (MFI) of FOXP3 staining. **B**. The suppressive activity of ESO-specific and control polyclonal populations was assessed by co-culture with CFSE-labeled conventional CD4^+^ T cells, at a responder:suppressor ratio of 1∶1, in the presence of irradiated monocytes and PHA. Dot plots show the CFSE-dilution profile in the absence of test population (left) and in the presence of the indicated test populations. Numbers in histograms correspond the percentage of undivided cells. Results corresponding to the calculated% suppression are shown for all tested populations.

One caveat of the experimental setting used above was that, whereas ESO-specific cells were detected based on their capacity to specifically secrete IFN-γ in response to ESO, Treg secreted little IFN-γ even upon stimulation with PMA/ionomycin (Figure 2). To overcome the limitations imposed by the detection of ESO-specific CD4^+^ T cells based on functional assays, we have recently developed soluble fluorescent MHC class II tetramers that incorporate an immunodominant epitope from ESO, corresponding to peptide 119–143 [17], [18]. We have shown that MHC class II/ESO_119–143_ tetramers stain ESO-specific CD4^+^ T cells in circulating lymphocytes of patients immunized with an ESO recombinant vaccine with high efficiency and specificity, allowing their direct visualization among polyspecific CD4^+^ T-cell cultures. Because 4 patients with spontaneous immune responses to ESO expressed MHC class II alleles for which the suitable tetramers were available (DR52b and DR4), we assessed ESO-specific T cells in these cultures by tetramer staining (Figure 4C and 4D). We detected significant proportions of ESO tetramer^+^ cells in cultures from CD25^−^CD127^+^ populations from all patients as well as in those from the CD25^−^CD127^−^ populations (in 3 patients at significantly increased frequency as compared to the CD25^−^CD127^+^ fractions). However, in all cases, we failed to detect significant proportions of tetramers^+^ cells in Treg cultures. In addition to CD25^+^CD127^−^FOXP3^+^Treg, suppressive CD4^+^ T cells have also been described in some studies among other populations [21]. To assess if, regardless of their phenotype, ESO-specific CD4^+^ T cells displayed suppressive functions, we isolated them from the cultures by tetramer- or IFN-γ-guided cell sorting and assessed them functionally in a suppression assay as described [23], [24]. As expected, FOXP3^+^Treg populations simultaneously assessed as internal controls efficiently suppressed the growth of responder cells. In contrast, ESO-specific cells isolated from both CD25^−^CD127^+^ and CD25^−^CD127^+^ cultures were FOXP3^−^ and were not suppressive (Figure 5).

### *Ex vivo* assessment of ESO-specific circulating CD4^+^ T cells using MHC class II/ESO tetramers

**Figure 6:**
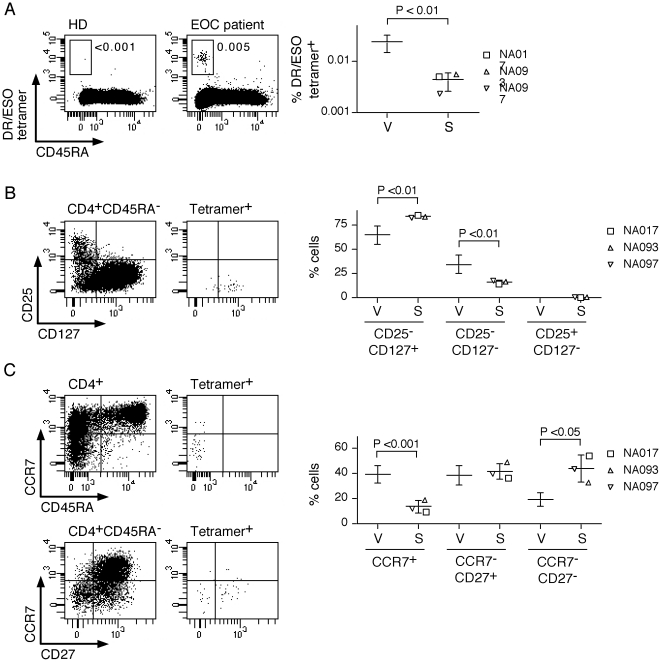
*Ex vivo* assessment of ESO-specific CD4^+^ T cells using DR52b/ESO tetramers. CD4^+^ T cells from DR52b^+^ healthy donors (HD) and patients were stained *ex vivo* with DR52b/ESO_119–143_ tetramers and mAb specific for CD45RA, CD25, CD127, CCR7 and CD27 and analyzed by flow cytometry. **A**. Dot plots for one HD and one EOC patient are shown. Numbers in dot plots correspond to the percentage of tetramer^+^ cells among CD45RA^−^ memory cells. Data for all EOC patients with spontaneous immune responses to ESO (S) are shown in comparison to the frequency (mean ± SD) of ESO tetramer^+^ cells in post-vaccine samples from patients having received a recombinant ESO vaccine (V) [17]. **B**. Dot plots show the expression of CD25 and CD127 in total memory cells and in tetramer^+^ cells of one EOC patient. Data corresponding to the proportion of conventional, CD25^−^CD127^+^ and CD25^−^CD127^−^, and Treg, CD25^+^CD127^−^, populations within tetramer^+^ cells for all EOC patients (S) are summarized and compared to the proportion (mean ± SD) of these populations in vaccine-induced tetramer^+^ cells (V). **C**. Dot plots show the expression of CCR7 and CD27 in total memory cells and in tetramer^+^ cells of one EOC patient. Data corresponding to the proportion of CCR7^+^, CCR7^−^CD27^+^ and CCR7^−^CD27^−^ populations within tetramer^+^ cells for all EOC patients (S) are summarized and compared to the proportion (mean ± SD) of these populations in vaccine-induced tetramer^+^ cells (V). Statistical analyses were performed using a standard two-tailed *t*-test.

To confirm the distribution of ESO-specific cells in CD4^+^ T-cell subsets in patients with spontaneous immune responses and further assess their proportions and phenotype, we stained total circulating CD4^+^ T cells from DR52b^+^ patients *ex vivo* with ESO tetramers in combination with mAb directed against markers that characterize distinct differentiation stages of CD4^+^ T cells. As shown previously [17], ESO tetramer^+^ cells were not detectable in CD4^+^ T cells from healthy donors. In contrast, they were clearly detected *ex vivo* in circulating memory CD4^+^ T cells from the patients (Figure 6A). Tetramer^+^ cells in circulating CD4^+^ T cells from the patients were uniformly CD25^−^ and were found among both CD127^+^ and CD127^−^ populations, but were not detectable among CD25^+^CD127^−^ Treg (Figure 6B). Thus, similar to what we have previously observed in patients immunized with a recombinant ESO vaccine, direct *ex vivo* staining with ESO tetramers confirmed the lack of significant proportions of ESO-specific CD4^+^ T cells in circulating Treg of patients with spontaneous immunological responses to the antigen. The frequency of circulating ESO tetramers^+^ cells in the patients with spontaneous responses, was, in average, of 1∶25000, five folds lower than that found in vaccinated patients (Figure 6A) [17]. However, ESO tetramer^+^ cells in patients with spontaneous responses contained lower proportions of CD127^−^ cells as compared to those of vaccinated patients (Figure 6B). In addition, in contrast to the latter, which contained, in average, roughly comparable proportions of CCR7^+^ and CCR7^−^ cells, as well as a large majority of CD27^+^ cells, ESO tetramer^+^ cells in patients with spontaneous responses were mostly CCR7^−^ and contained increased proportions of CD27^−^ cells, consistent with a more differentiated phenotype (Figure 6C). Finally, to exclude the possibility that the selection of ESO Ab^+^ patients may have selected for patients with low or absent ESO-specific Treg, we assessed *ex vivo* CD4^+^ T cells from ESO Ab^−^ DR52b^+^ patients (n = 23). However, we failed to detect significant levels of ESO tetramer^+^ in these patients (data not shown).

## Discussion

Strong immune responses to cancerous tissues are potentially able to prevent disease progression. Because these responses, often directed not exclusively against tumor-specific antigens, but also against differentiation or other self-antigens, have the potential to considerably damage normal tissues, robust immunoregulatory networks are in place [25]. In particular, CD4^+^CD25^+^FOXP3^+^Treg, involved in maintaining tissue homeostasis and self-tolerance, are believed to play a major role in dampening anticancer immunity. Previous studies have reported increased proportions of Treg in the circulation of at least part of patients with solid tumors [8], [9] as well as accumulation of CD25^+^FOXP3^+^Treg at tumor sites [8], [26], particularly at advanced stages of disease, and have established a correlation between their frequency and clinical outcomes [3]. On the base of these findings, approaches for eliminating Treg have been designed, and are already being assessed in clinical settings, although they are linked with the risk of unleashing auto-reactivity [27], [28], [29].

In this study, we have addressed the presence and distribution of CD4^+^ T cells specific for the tumor antigen ESO, of the cancer/testis group [10], [30], [31], within circulating CD4^+^ T-cell subsets of EOC patients. We used a combination of functional approaches and MHC class II tetramers incorporating an immunodominant peptide, ESO_119–143_, that we have recently developed [17], [18]. We found no significant differences in the proportion of Treg among circulating lymphocytes of EOC patients as compared with healthy donors. Similarly, we found no significant increase in the proportion of total circulating CD4^+^ CD25^−^CD127^−^ T cells, a population that contains recently activated CD4^+^ T cells and has been proposed to contain IL-10-secreting Tr1 cells in healthy donors [21] but had not been assessed in cancer patients prior to this study. Thus, whereas alterations in the circulating Treg compartment may indeed occur in some cancer patients, we found no major alterations in our group of EOC patients. It is noteworthy, however, that the patients assessed in the present study had received first line chemotherapy, which may temporarily reduce Treg [32], although they were assessed at various times, but at least one month, after termination of treatment. However, assessment of the immune status of this population, with respect to anti-tumor responses, is most relevant for the design of immune-based treatments.

By assessing ESO-specific responses in circulating CD4^+^ T-cell subsets from EOC patients with spontaneous immune responses, sorted *ex vivo* and stimulated *in vitro* with ESO peptides, we detected significant proportions of cells producing IFN-γ in response to ESO, but no IL-10- or IL-17-secreting cells, indicating that the majority of ESO-specific CD4^+^ T cells were T_H_1 effectors. Because this study specifically addressed T_H_1 cells and Treg in EOC, we limited the analysis to the most relevant cytokines, namely IFN-γ, IL-10 and, because of the reported relationship between Treg and T_H_17 cells, IL-17. It will however be of interest, in future studies, to address the production of additional cytokines such as IL-4 or IL-13, by ESO specific CD4^+^ T cells. In line with the conclusion that ESO-specific CD4^+^ T cells isolated from the cultures are effector cells, they did not exhibit significant suppressive functions. In addition, we did not detect ESO-specific cells in circulating Treg of patients with spontaneous immune responses to the antigen by staining, with MHC class II/ESO tetramers, of defined conventional and Treg CD4^+^ T-cell subpopulations isolated *ex vivo* by flow cytometry cell sorting, as well as by direct *ex vivo* staining with tetramers. At variance with our data, previous studies have reported the isolation of ESO-specific CD4^+^ T cells with suppressive functions from circulating lymphocytes of patients with advanced melanoma [19], [33]. Thus, whereas our data do not exclude the existence of ESO-specific Treg, they imply that the latter are not commonly found in circulating CD4^+^ T cells from EOC patients. It is also noteworthy that, consistent with our results, studies in mouse models have examined the presence of Treg in antigen-specific T cells with tetramers and failed to detect tetramer-binding Treg [34], [35].

Using MHC class II/ESO_119-143_ tetramers *ex vivo*, we could directly compare ESO-specific CD4^+^ T cells spontaneously arising in patients, with those induced through vaccination with a recombinant ESO protein (rESO) administered with Montanide™ and CpG [16]. It is noteworthy that until the recent development of MHC class II/peptide tetramers, it has been difficult to assess antigen-specific CD4^+^ T cells *ex vivo* due to their generally low frequency. This is indeed the first report characterizing CD4^+^ T cells specific for a cancer/testis antigen in cancer patients with spontaneous immune responses *ex vivo.* We found that the frequency of CD4^+^ T cells specific for ESO was significantly lower in patients with spontaneous immune responses as compared to that found in vaccinated patients. In addition, the phenotype of naturally-arising ESO tetramer^+^ cells was more differentiated, as compared to their vaccine-induced counterparts, containing increased proportions of CCR7^−^ and CD27^−^ cells. Previous studies exploring the correlation between the quality of memory CD4^+^ T-cell responses elicited by pathogens or vaccines and their phenotype, have suggested that a protective memory response should include not only effectors (CCR7^−^) but memory cells at earlier differentiation stages, namely central memory (CCR7^+^) and transitional memory (CCR7^−^CD27^+^) T cells [36], [37]. Based on this concept, our results suggest that ESO-specific CD4^+^ T cells induced through immunization of cancer patients with the rESO vaccine are likely to be not only quantitatively but also qualitatively superior to those arising during spontaneous immune responses, which further encourages the use of ESO-based vaccines in patients bearing ESO-expressing tumors.

Together, these results underline the potential of MHC class II/ESO tetramers for the unambiguous detection, quantification and phenotyping of ESO-specific CD4^+^ T cells, not only following vaccination, but also for assessing the immune responses that naturally arise in patients bearing antigen-expressing tumors. The further application of this approach for the characterization of CD4^+^ T cells specific for ESO and other tumor antigens in the circulation and at tumor sites of cancer patients, both along the natural course of the disease and during therapy, will likely contribute to significantly further our understanding of their roles and contribution in immunity to cancer.

## Materials and Methods

### Patient and healthy donor samples and assessment of ESO-specific antibody responses

Sera and peripheral blood mononuclear cells (PBMC) were collected from EOC patients seen at CLCC René Gauducheau and from healthy individuals upon written informed consent and approval by the Institutional Review Board (Comité de Protection des Personnes Ouest IV – Nantes). Antibody (Ab) responses to ESO were assessed by ELISA, as previously described [14], [16], using recombinant ESO protein (rESO) produced in E. coli.

### *Ex vivo* phenotypic assessment of CD4^+^ T-cell subsets and flow cytometry cell sorting

CD4^+^ T cells were enriched by positive selection from PBMC of healthy individuals and EOC patients by magnetic cell sorting (Miltenyi Biotec), stained with monoclonal antibodies (mAb) specific for CD4 (BD Biosciences), CD8 (BD Biosciences), CD45RA (BD Biosciences), CD25 (Beckman Coulter), CD127 (eBioscience) and FOXP3 (eBioscience), as indicated, and analyzed by flow cytometry using an LSRII (BD Biosciences). For *ex vivo* flow cytometry cell sorting, enriched CD4^+^ T cells were stained with anti-CD4, -CD8, -CD45RA, -CD25 and -CD127 mAb. After gating on CD4^+^CD8^−^CD45RA^−^ lymphocytes, cells were separated into memory CD25^−^CD127^+^, CD25^−^CD127^−^ and CD25^+^CD127^−^Treg populations to high purity (>97%) using a FACSAria (BD Biosciences).

### *In vitro* stimulation and functional assessment of CD4^+^ T-cell subsets

Total CD4^+^ T cells or *ex vivo* sorted memory conventional and Treg CD4^+^ T-cell populations were stimulated *in vitro* with either a pool of long overlapping peptides covering the ESO sequence [16] or with anti-CD2/3/28-coated microbeads (Miltenyi Biotec) in the presence of irradiated autologous APC and were cultured in the presence of recombinant human IL-2 (Chiron). Day 12 to 14 cultures were assessed in a 4-h intracellular cytokine staining assay using mAb specific for IFN-γ (BD Biosciences), IL-10 (BD Biosciences) and IL-17 (eBioscience), following stimulation with either the ESO peptide pool or PMA (100 ng/mL, Sigma Aldrich) and ionomycin (1 µg/mL, Sigma Aldrich), as indicated, and analyzed by flow cytometry (LSRII).

### MHC class II/ESO peptide tetramer staining

Fluorescent HLA-DR52b/and HLA-DR4/ESO_119–143_ tetramers were generated as previously described [17], [18]. ESO-stimulated CD4^+^ T-cell cultures were incubated with tetramers at a final concentration of 3 µg/mL for 1 h at 37°C and then stained with CD4-specific mAb and analyzed by flow cytometry (LSRII). For *ex vivo* enumeration and phenotyping of specific cells, total CD4^+^ T cells enriched from PBMC by magnetic cell sorting were rested overnight, incubated with tetramers (3 µg/mL) for 2 h at 37°C and then stained with the indicated mAb and analyzed by flow cytometry (LSRII).

### Isolation of ESO-specific polyclonal populations and assessment of suppressive function

ESO-specific T cells were isolated from peptide-stimulated cultures by IFN-γ secretion assay- (Miltenyi Biotec) or tetramer-guided flow cytometry cell sorting and expanded by stimulation with PHA and irradiated allogeneic PBMC in the presence of IL-2, as previously described [16], [17]. The specificity of the obtained polyclonal cultures was assessed by tetramer staining or by intracellular IFN-γ staining following restimulation with ESO peptides and FOXP3 expression was assessed by staining using specific mAb. The suppressive activity of ESO-specific polyclonal cultures was assessed by co-culture of CFSE-labeled responder conventional CD4^+^ T cells with or without test populations in the presence of irradiated monocytes, enriched by positive selection from PBMC by magnetic cell sorting (Miltenyi Biotec), and PHA, as previously described [23], [24]. Growth of responder cells was assessed by flow cytometry analysis of CFSE dilution in day 4 to 6 cultures. The growth (100-% undivided cells) of the wells with suppressor cells (experimental group) was compared with that of the wells without suppressors (control). The percentage of suppression was determined as follows: 100 – ((growth of experimental group/growth of control) ×100).

## References

[1] Kennedy R, Celis E (2008). Multiple roles for CD4+ T cells in anti-tumor immune responses.. Immunol Rev.

[2] Muranski P, Restifo NP (2009). Adoptive immunotherapy of cancer using CD4(+) T cells.. Curr Opin Immunol.

[3] Curiel TJ, Coukos G, Zou L, Alvarez X, Cheng P (2004). Specific recruitment of regulatory T cells in ovarian carcinoma fosters immune privilege and predicts reduced survival.. Nat Med.

[4] Sato E, Olson SH, Ahn J, Bundy B, Nishikawa H (2005). Intraepithelial CD8+ tumor-infiltrating lymphocytes and a high CD8+/regulatory T cell ratio are associated with favorable prognosis in ovarian cancer.. Proc Natl Acad Sci U S A.

[5] Hiraoka N, Onozato K, Kosuge T, Hirohashi S (2006). Prevalence of FOXP3+ regulatory T cells increases during the progression of pancreatic ductal adenocarcinoma and its premalignant lesions.. Clin Cancer Res.

[6] Gao Q, Qiu SJ, Fan J, Zhou J, Wang XY (2007). Intratumoral balance of regulatory and cytotoxic T cells is associated with prognosis of hepatocellular carcinoma after resection.. J Clin Oncol.

[7] Kobayashi N, Hiraoka N, Yamagami W, Ojima H, Kanai Y (2007). FOXP3+ regulatory T cells affect the development and progression of hepatocarcinogenesis.. Clin Cancer Res.

[8] Woo EY, Yeh H, Chu CS, Schlienger K, Carroll RG (2002). Cutting edge: Regulatory T cells from lung cancer patients directly inhibit autologous T cell proliferation.. J Immunol.

[9] Liyanage UK, Moore TT, Joo HG, Tanaka Y, Herrmann V (2002). Prevalence of regulatory T cells is increased in peripheral blood and tumor microenvironment of patients with pancreas or breast adenocarcinoma.. J Immunol.

[10] Chen YT, Scanlan MJ, Sahin U, Tureci O, Gure AO (1997). A testicular antigen aberrantly expressed in human cancers detected by autologous antibody screening.. Proc Natl Acad Sci U S A.

[11] Valmori D, Dutoit V, Liénard D, Rimoldi D, Pittet M (2000). Naturally occurring HLA-A2 restricted CD8+ T cell response to the cancer testis antigen NY-ESO-1 in melanoma patients.. Cancer Res.

[12] Cheever MA, Allison JP, Ferris AS, Finn OJ, Hastings BM (2009). The prioritization of cancer antigens: a national cancer institute pilot project for the acceleration of translational research.. Clin Cancer Res.

[13] Jäger E, Chen YT, Drijfhout JW, Karbach J, Ringhoffer M (1998). Simultaneous humoral and cellular immune response against cancer-testis antigen NY-ESO-1: definition of human histocompatibility leukocyte antigen (HLA)-A2-binding peptide epitopes.. J Exp Med.

[14] Valmori D, Souleimanian NE, Hesdorffer CS, Ritter G, Old LJ (2005). Identification of B cell epitopes recognized by antibodies specific for the tumor antigen NY-ESO-1 in cancer patients with spontaneous immune responses.. Clin Immunol.

[15] Ayyoub M, Souleimanian NE, Godefroy E, Scotto L, Hesdorffer CS (2006). A phenotype based approach for the immune monitoring of NY-ESO-1 specific CD4+ T cell responses in cancer patients.. Clin Immunol.

[16] Valmori D, Souleimanian NE, Tosello V, Bhardwaj N, Adams S (2007). Vaccination with NY-ESO-1 protein and CpG in Montanide induces integrated antibody/Th1 responses and CD8 T cells through cross-priming.. Proc Natl Acad Sci U S A.

[17] Ayyoub M, Dojcinovic D, Pignon P, Raimbaud I, Schmidt J (2010). Monitoring of NY-ESO-1 specific CD4+ T cells using molecularly defined MHC class II/His-tag-peptide tetramers.. Proc Natl Acad Sci U S A.

[18] Ayyoub M, Pignon P, Dojcinovic D, Raimbaud I, Old LJ (2010). Assessment of vaccine-induced CD4 T cell responses to the 119-143 immunodominant region of the tumor-specific antigen NY-ESO-1 using DRB1*0101 tetramers.. Clin Cancer Res.

[19] Fourcade J, Sun Z, Kudela P, Janjic B, Kirkwood JM (2010). Human tumor antigen-specific helper and regulatory T cells share common epitope specificity but exhibit distinct T cell repertoire.. J Immunol.

[20] Nicholaou T, Ebert LM, Davis ID, McArthur GA, Jackson H (2009). Regulatory T-cell-mediated attenuation of T-cell responses to the NY-ESO-1 ISCOMATRIX vaccine in patients with advanced malignant melanoma.. Clin Cancer Res.

[21] Haringer B, Lozza L, Steckel B, Geginat J (2009). Identification and characterization of IL-10/IFN-gamma-producing effector-like T cells with regulatory function in human blood.. J Exp Med.

[22] Odunsi K, Jungbluth AA, Stockert E, Qian F, Gnjatic S (2003). NY-ESO-1 and LAGE-1 cancer-testis antigens are potential targets for immunotherapy in epithelial ovarian cancer.. Cancer Res.

[23] Valmori D, Merlo A, Souleimanian NE, Hesdorffer CS, Ayyoub M (2005). A peripheral circulating compartment of natural naive CD4 Tregs.. J Clin Invest.

[24] Valmori D, Raffin C, Raimbaud I, Ayyoub M (2010). Human ROR{gamma}t+ TH17 cells preferentially differentiate from naive FOXP3+Treg in the presence of lineage-specific polarizing factors.. Proc Natl Acad Sci U S A.

[25] Mougiakakos D, Choudhury A, Lladser A, Kiessling R, Johansson CC (2010). Regulatory T cells in cancer.. Adv Cancer Res.

[26] Woo EY, Chu CS, Goletz TJ, Schlienger K, Yeh H (2001). Regulatory CD4(+)CD25(+) T cells in tumors from patients with early-stage non-small cell lung cancer and late-stage ovarian cancer.. Cancer Res.

[27] Maker AV, Phan GQ, Attia P, Yang JC, Sherry RM (2005). Tumor regression and autoimmunity in patients treated with cytotoxic T lymphocyte-associated antigen 4 blockade and interleukin 2: a phase I/II study.. Ann Surg Oncol.

[28] Attia P, Phan GQ, Maker AV, Robinson MR, Quezado MM (2005). Autoimmunity correlates with tumor regression in patients with metastatic melanoma treated with anti-cytotoxic T-lymphocyte antigen-4.. J Clin Oncol.

[29] Lute KD, May KF, Lu P, Zhang H, Kocak E (2005). Human CTLA4 knock-in mice unravel the quantitative link between tumor immunity and autoimmunity induced by anti-CTLA-4 antibodies.. Blood.

[30] Simpson AJ, Caballero OL, Jungbluth A, Chen YT, Old LJ (2005). Cancer/testis antigens, gametogenesis and cancer.. Nat Rev Cancer.

[31] Caballero OL, Chen YT (2009). Cancer/testis (CT) antigens: potential targets for immunotherapy.. Cancer Sci.

[32] Wu X, Feng QM, Wang Y, Shi J, Ge HL (2010). The immunologic aspects in advanced ovarian cancer patients treated with paclitaxel and carboplatin chemotherapy.. Cancer Immunol Immunother.

[33] Vence L, Palucka AK, Fay JW, Ito T, Liu YJ (2007). Circulating tumor antigen-specific regulatory T cells in patients with metastatic melanoma.. Proc Natl Acad Sci U S A.

[34] Antunes I, Tolaini M, Kissenpfennig A, Iwashiro M, Kuribayashi K (2008). Retrovirus-specificity of regulatory T cells is neither present nor required in preventing retrovirus-induced bone marrow immune pathology.. Immunity.

[35] Burchill MA, Yang J, Vang KB, Moon JJ, Chu HH (2008). Linked T cell receptor and cytokine signaling govern the development of the regulatory T cell repertoire.. Immunity.

[36] Okoye A, Meier-Schellersheim M, Brenchley JM, Hagen SI, Walker JM (2007). Progressive CD4+ central memory T cell decline results in CD4+ effector memory insufficiency and overt disease in chronic SIV infection.. J Exp Med.

[37] Riou C, Yassine-Diab B, Van grevenynghe J, Somogyi R, Greller LD (2007). Convergence of TCR and cytokine signaling leads to FOXO3a phosphorylation and drives the survival of CD4+ central memory T cells.. J Exp Med.

